# Renal Venous Stasis Index Reflects Renal Congestion and Predicts Adverse Outcomes in Patients With Heart Failure

**DOI:** 10.3389/fcvm.2022.772466

**Published:** 2022-03-07

**Authors:** Himika Ohara, Akiomi Yoshihisa, Yuko Horikoshi, Shinji Ishibashi, Mitsuko Matsuda, Yukio Yamadera, Yukiko Sugawara, Yasuhiro Ichijo, Yu Hotsuki, Koichiro Watanabe, Yu Sato, Tomofumi Misaka, Takashi Kaneshiro, Masayoshi Oikawa, Atsushi Kobayashi, Yasuchika Takeishi

**Affiliations:** ^1^Department of Cardiovascular Medicine, Fukushima Medical University, Fukushima, Japan; ^2^Department of Clinical Laboratory Sciences, Fukushima Medical University School of Health Science, Fukushima, Japan; ^3^Department of Clinical Laboratory Medicine, Fukushima Medical University, Fukushima, Japan

**Keywords:** heart failure, renal circulation, hemodynamics, kidney, prognosis

## Abstract

**Background:**

It has been recently reported that the renal venous stasis index (RVSI) assessed by renal Doppler ultrasonography provides information to stratify pulmonary hypertension that can lead to right-sided heart failure (HF). However, the clinical significance of RVSI in HF patients has not been sufficiently examined. We aimed to examine the associations of RVSI with parameters of cardiac function and right heart catheterization (RHC), as well as with prognosis, in patients with HF.

**Methods:**

We performed renal Doppler ultrasonography, echocardiography and RHC in hospitalized patients with HF (*n* = 388). RVSI was calculated as follows: RVSI = (cardiac cycle time-venous flow time)/cardiac cycle time. The patients were classified to three groups based on RVSI: control group (RVSI = 0, *n* = 260, 67%), low RVSI group (0 < RVSI ≤ 0.21, *n* = 63, 16%) and high RVSI group (RVSI > 0.21, *n* = 65, 17%). We examined associations of RVSI with parameters of cardiac function and RHC, and followed up for cardiac events defined as cardiac death or worsening HF.

**Results:**

There were significant correlations of RVSI with mean right atrial pressure (mRAP; *R* = 0.253, *P* < 0.001), right atrial area (*R* = 0.327, *P* < 0.001) and inferior vena cava diameter (*R* = 0.327, *P* < 0.001), but not with cardiac index (*R* = −0.019, *P* = 0.769). During the follow-up period (median 412 days), cardiac events occurred in 60 patients. In the Kaplan–Meier analysis, the cumulative cardiac event rate increased with increasing RVSI (log-rank, *P* = 0.001). In the multivariate Cox proportional hazard analysis, the cardiac event rate was independently associated with RVSI (high RVSI group vs. control group: hazard ratio, 1.908; 95% confidence interval, 1.046–3.479, *P* = 0.035).

**Conclusion:**

RVSI assessed by renal Doppler ultrasonography reflects right-sided overload and is associated with adverse prognosis in HF patients.

## Introduction

Heart failure (HF) is a refractory clinical syndrome that originates from various types of structural or functional heart diseases. The number of patients with HF has been rapidly increasing, and HF is becoming a major public health concern worldwide (1, 2). Congestion is a key feature in HF, and its presence is associated with poor prognosis. However, congestion can go unrecognized, as it is sometimes not clinically evident. To evaluate the degree of congestion objectively, non-invasive image testing is required in patients with HF (3–5).

It has already been reported that the intrarenal venous flow (IRVF) patterns assessed by renal Doppler ultrasonography were associated with congestion and prognosis in patients with HF. Specifically, the most adverse prognosis was present in HF patients with monophasic IRVF pattern, followed in order by biphasic IRVF pattern and continuous IRVF pattern (6–9). However, the classification of IRVF patterns is not necessarily clear. There are patterns for which classification is difficult or classification itself may miss important changes in the pattern. Renal venous stasis index (RVSI) is a novel indicator of cardiac cycle-dependent stasis of renal venous flow. RVSI indicates the proportion of the cardiac cycle during which there is no renal venous outlet flow. RVSI can quantify IRVF patterns and completement the weaknesses of the IRVF pattern classification. According to a past study, RVSI provided information to stratify prognosis in patients with pulmonary hypertension (PH) in terms of propensity to develop right-sided HF. Namely, PH patients with high RVSI experienced more adverse events such as worsening PH or all-cause mortality (10). However, the clinical significance of RVSI in HF patients has not been sufficiently examined. We aimed to elucidate the associations of RVSI with parameters of cardiac function and right heart catheterization (RHC), as well as with cardiac events defined as cardiac death and worsening HF, in patients with HF.

## Methods

### Subjects and Protocol

This was a prospective observational study of 402 patients who were classified as either stage C or stage D of heart failure stage classification in the American College of Cardiology Foundation/ American Heart Association guideline and were hospitalized to Fukushima Medical University Hospital between April 2018 and September 2020 (11). Treatment of decompensated HF was provided by each patient's attending cardiologist based on the established HF guidelines (11, 12). Blood samples, renal Doppler ultrasonography and echocardiography were obtained during hospitalization in the patients in a stable condition before discharge. We subsequently excluded patients with who were undergoing dialysis (*n* = 14). At last, 388 patients were enrolled in this study. Two hundred and forty five of them had undergone RHC within 3 days of renal Doppler ultrasonography. Of these 388 patients, patients with RVSI of 0 were defined as the control group (*n* = 260, 67%). Patients with RVSI above 0 were divided into two groups on the basis of the median value of RVSI (0.21): the low RVSI group (0 < RVSI ≤ 0.21, *n* = 63, 16%) and the high RVSI group (RVSI > 0.21, *n* = 65, 17%).

First, we compared the clinical features as well as the results from laboratory tests, echocardiography and RHC between the three groups. Second, the patients were followed up until November 2020 for cardiac events defined as cardiac death or worsening HF. Cardiac death was defined as death from acute coronary syndrome, ventricular fibrillation and HF, and worsening HF was defined as unplanned re-hospitalization for HF treatment. For patients who experienced ≥ two events, only the first event was included in the analysis. These patients visited hospital monthly or every other month. Therefore, we could follow up on all patients. Disease status and dates of death were gained from the patient's medical records. The results of the analysis were hidden from those conducting the survey, and written informed consent was gained from all enrolled patients. The protocol for this study was approved by the Ethics Committee of Fukushima Medical University and was conducted in accordance with the principles described in the Declaration of Helsinki. We reported this study in conformity to Strengthening the Reporting of Observational Studies in Epidemiology and the Enhancing the Quality and Transparency of Health Research guidelines.

Ischemic coronary artery disease was confirmed by either myocardial scintigraphy or coronary computed tomography angiography and/or coronary angiography (13). Atrial fibrillation (AF) was confirmed by electrocardiogram performed during hospitalization or from medical records including past medical history. Hypertension was defined as systolic blood pressure of ≥ 140 mmHg, diastolic blood pressure of ≥ 90 mmHg, or taking antihypertensive drugs. Dyslipidemia was defined as levels of triglyceride ≥ 150 mg/dL, levels of low-density lipoprotein cholesterol ≥ 140 mg/dL, levels of high-density lipoprotein cholesterol <40 mg/dL, or taking cholesterol-lowering drugs. Diabetes mellitus was defined as recent use of antidiabetic drugs, levels of fasting glucose ≥ 126 mg/dL, levels of casual glucose ≥ 200 mg/dL and/or levels of HbA1c ≥ 6.5% (National Glycohemoglobin Standardization Program) (14). Chronic kidney disease (CKD) was defined as estimated glomerular filtration rate (eGFR) of <60 mL/min per 1.73 m^2^ (15–17). Anemia was defined as levels of hemoglobin <12.0 g/dL in female and <13.0 g/dL in male (18, 19).

### Renal Doppler Ultrasonography

**Figure 1:**
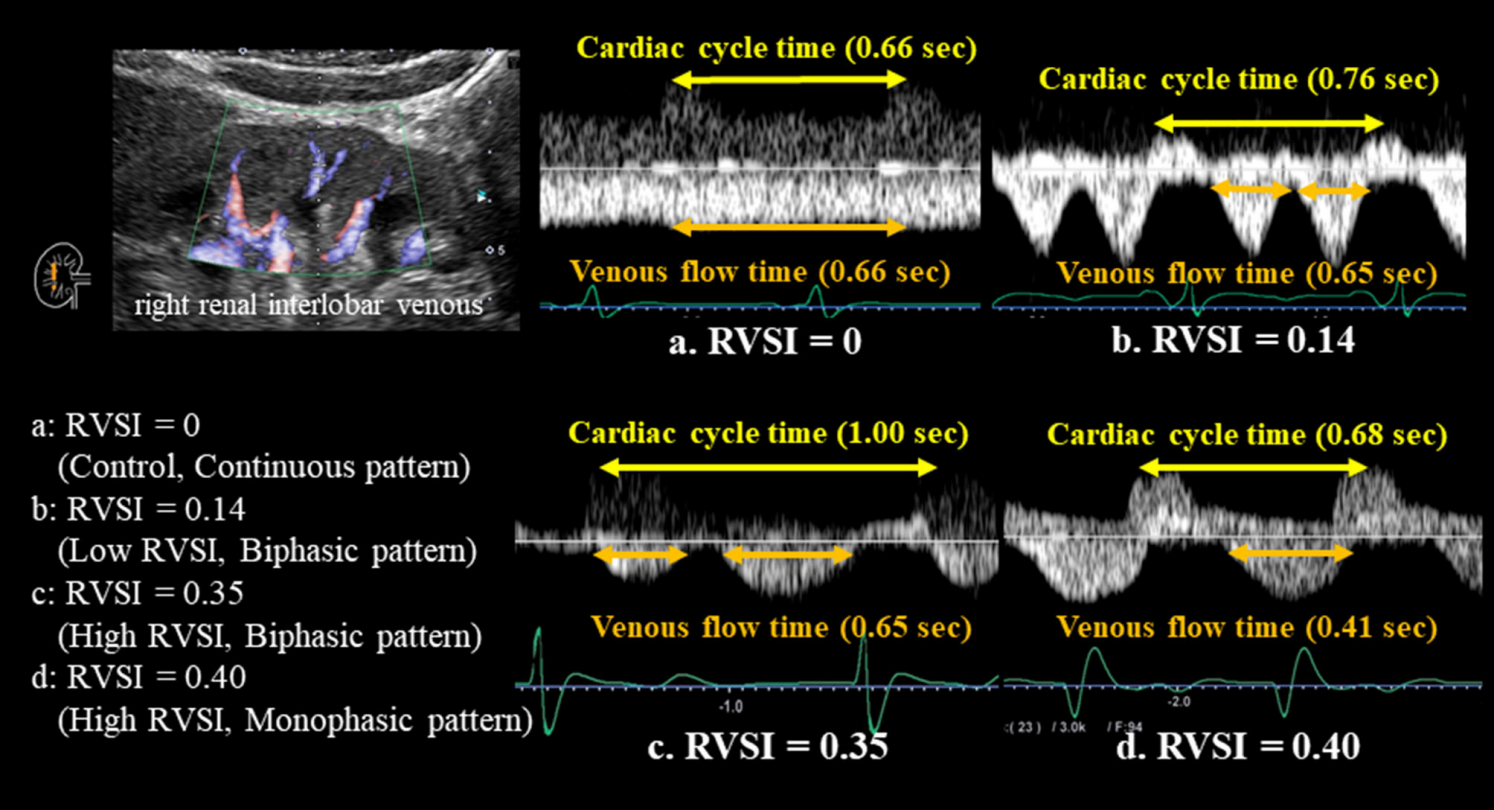
All renal Doppler ultrasonography studies were performed in the right kidney. Color Doppler images were used to record pulsed Doppler waveforms of the interlobar arteries and veins simultaneously. The upward Doppler signal indicated the intrarenal arterial flow, and the downward Doppler signal indicated the venous flow. We measured venous flow time and calculated RVSI as follows: (cardiac cycle time-venous flow time)/cardiac cycle time. RVSI of 0 was defined as the control. RVSI above 0 were divided based on the median value of RVSI (0.21): the low RVSI (0 < RVSI ≤ 0.21) and the high RVSI (RVSI > 0.21). RVSI increased with increasing severity of renal congestion. This figure shows representative IRVF patterns with RVSI in a patient with HF. IRVF patterns were broadly categorized into continuous (RVSI = 0, non-congestive) and discontinuous (RVSI > 0, nadir velocity = 0) flow patterns. We further classified the discontinuous IRVF patterns into two stages: biphasic (with venous peaks during systole and diastole), and monophasic (with venous peak during diastole). **(a)** RVSI = 0 (control) with continuous pattern; **(b)** low RVSI with biphasic pattern; **(c)** high RVSI with biphasic pattern; and **(d)** high RVSI with monophasic pattern. RVSI, renal venous stasis index; IRVF, intrarenal venous flow.

The actual methods of acquisition were as follows. The patients were performed renal Doppler ultrasonography in a stable condition after treatment. Two experienced sonographers (M.M, with 27 years of experience in abdominal ultrasonography, and S.I, with 19 years of experience) performed renal Doppler ultrasonography, using an Aplio i800 system (Canon Medical Systems Corporation, Tochigi, Japan) with a convex transducer frequency range of 2.5–5.0 MHz. The velocity range of the color Doppler was set to approximately 10–20 cm/s. The two examiners were blinded to all clinical data. The patients fasted for at least 12 h before the renal Doppler ultrasonography and were placed in the lateral position. All renal Doppler ultrasonography examinations were performed in the right renal vein. This was because left renal vein is entrapped in the fork between the abdominal aorta and the superior mesenteric artery, thus attenuating its phasicity. Furthermore, although this is a rare event in cases of ovarian or testicular varicose veins, the left ovarian or testicular veins draining into the left renal vein may have affected renal venous flow. The transducer was placed in the lateral abdominal region, and the patient's arms were raised above the chest to obtain a proper acoustic window. The patients were holding their breath while measurements. As shown in Figure 1, we used color Doppler images to record pulsed Doppler waveforms of the interlobar arteries and veins simultaneously. Transducer was placed with an angle of <60 degrees. The upward Doppler signal indicates the intrarenal arterial flow, and the downward Doppler signal indicates the venous flow. We used the interlobar vein flow to calculate RVSI. In patients with sinus rhythm, we selected the most stable value among five cardiac cycles. In patients with AF, an index beat (the beat following two preceding cardiac cycles of equal duration) was used for each measurement. We calculated RVSI, which indicates the proportion of the cardiac cycle during which there was no renal venous outlet flow, as follows: (cardiac cycle time - venous flow time)/cardiac cycle time (10). IRVF patterns were broadly categorized into continuous (RVSI = 0, non-congestive) and discontinuous (RVSI > 0, nadir velocity = 0) flow patterns. We further classified the discontinuous IRVF patterns into two stages: biphasic (with venous peaks during systole and diastole), and monophasic (with venous peak during diastole) (7). Figure 1 shows representative RVSI along with IRVF patterns. Figure 1a, RVSI = 0 (control) with continuous pattern; Figure 1b, low RVSI with biphasic pattern; Figure 1c, high RVSI with biphasic pattern; and Figure 1d, high RVSI with monophasic pattern. Although both Figures 1b, c are classified into biphasic IRVF pattern, these patterns could be distinguished by RVSI (i.e., Figure 1b: Low RVSI and Figure 1c: High RVSI). On the contrary, both Figures 1c, d belonged to high RVSI, but classified into different IRVF patterns (i.e., Figure 1c: Biphasic pattern and Figure 1d: Monophasic pattern).

### Echocardiography

Echocardiography was performed by experienced echocardiographers based on current recommendations (20, 21). The echocardiographic parameters included left ventricular ejection fraction (LVEF), left ventricular outflow tract velocity-time integral (LVOT VTI), left atrial volume, early transmitral flow velocity to mitral annular velocity ratio (mitral valve E/e'), right atrium area, right ventricular areas, right ventricular fractional area change (RV-FAC), inferior vena cava (IVC) diameter, severity of tricuspid regurgitation (TR), tricuspid regurgitation pressure gradient (TRPG), tricuspid annular plane systolic excursion (TAPSE), TAPSE/systolic pulmonary artery pressure ratio (TAPSE/sPAP ratio), and tissue Doppler-derived tricuspid lateral annular systolic velocity (tricuspid valve S') (21). The LVEF was calculated by Simpson's method. The RV-FAC, defined as (end diastolic area - end systolic area)/end diastolic area × 100, is a measure of right ventricular systolic function. The TAPSE/sPAP ratio is an indicator of right ventricle–to–pulmonary circulation coupling (22–27). All measurements were performed using ultrasound systems (ACUSON Sequoia, Siemens Medical Solutions USA, Inc., Mountain View, CA, USA).

### Right Heart Catheterizations and Hemodynamic Measurements

Of the total of 388 patients, 245 underwent RHC based on remedial judgment of the attending physician. RHC was performed within 3 days of renal Doppler ultrasonography during hospitalization in the patients in a stable condition without changes in medications, including doses, similar to the setting for renal Doppler ultrasonography. All RHCs were performed under fluoroscopic guidance with the patients in the resting supine position breathing room air and at rest after more than 30 min of catheter placement. Cardiac output and mean right atrial pressure (mRAP) were measured using a 7F Swan-Ganz catheter (Edwards Lifesciences, Irvine, CA, USA). Cardiac output was calculated using the direct Fick method (28).

### Measurement of Laboratory Data

We measured B-type natriuretic peptide (BNP) levels using a specific immunoradiometric assay (Shionoria BNP kit, Shionogi, Osaka, Japan). This assay was performed by experienced laboratory technicians in blind.

### Statistical Analysis

Normally distributed data were presented as mean ± standard deviation, and non-normally distributed data were presented as median and interquartile range. Categorical variables were expressed as numbers and percentages, and the chi-square test was used for their comparisons. Among the three groups, parametric variables were compared using one-way analysis of variance, and non-parametric variables were compared using Kruskal-Wallis test. Kaplan-Meier analysis with log-rank test was used to evaluate the cardiac event rate. RVSI was assessed as a predictor of post-discharge cardiac events by the Cox proportional hazard analysis. In the univariate Cox proportional hazard analysis, to prepare for potential confounding, we considered the following clinical factors: age, sex and other confounding factors which differed statistically among the groups. Univariate factors with *P* < 0.05 were entered into the multivariate Cox proportional hazard analyses. A value of *P* < 0.05 was defined statistically significant for all comparisons. These analyses were performed using a statistical software package (SPSS ver. 27.0, IBM, Armonk, NY, USA).

## Results

**Figure 2:**
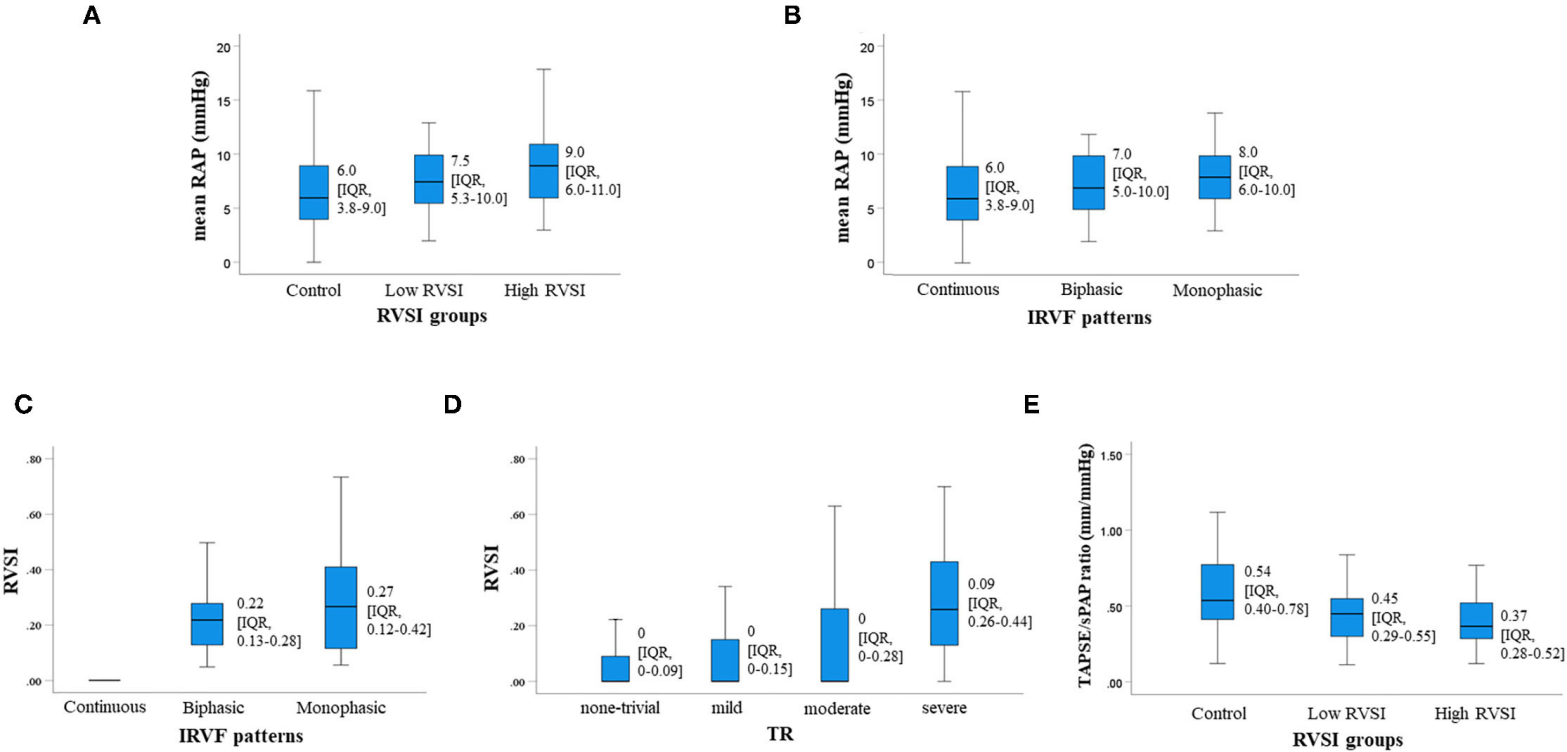
The severity of renal congestion can be assessed by measuring RVSI using renal Doppler ultrasonography. **(A,C–E)** show the relationship between RVSI and mRAP **(A)** IRVF patterns **(C)** severity of TR **(D)** and TAPSE/sPAP ratio **(E)**. RVSI showed a significant stepwise increase along the IRVF patterns (**C**; *P* < 0.001) and severity of tricuspid regurgitation (**D**; *P* < 0.001). TAPSE/sPAP ratio showed a significant stepwise decrease along the RVSI groups (**E**; *P* < 0.001). In addition, both RVSI and IRVF patterns showed similar associations with elevated mRAP (**A**; *P* < 0.001 and **B**; *P* = 0.001). RVSI, renal venous stasis index; mRAP, mean right atrial pressure; IRVF, intrarenal venous flow; TR, tricuspid regurgitation; TAPSE/sPAP, tricuspid annular plane systolic excursion/systolic pulmonary artery pressure; IQR, interquartile range.

Table 1 shows the clinical and demographic characteristics of the study population. Of the 388 patients included in the study, the median age was 71 (63.0–79.8) years and 225 (58.0%) were male. There were some unclassified IRVF patterns in our study. The prevalence of AF, hypertension, CKD and anemia increased in proportion to RVSI. In contrast, age, sex, body mass index, blood pressure, heart rate, coronary artery disease and diabetes mellitus, did not differ among the groups. There was not significant correlation between RVSI and heart rate (*R* = 0.009, *P* = 0.852). Regarding laboratory data, levels of BNP, C-reactive protein and urine albumin-to-creatinine ratio became higher, and levels of eGFR and hemoglobin became lower with increasing RVSI. However, sodium and proteinuria did not differ among the groups. With regard to the parameters of echocardiography, there was a significant increase in left atrial volume, mitral valve E/e', right atrial area, IVC diameter, severity of TR and TRPG, and a significant decrease in TAPSE and TAPSE/sPAP ratio with increasing RVSI. In contrast, LVEF and LVOT VTI were similar among the groups. Cardiopulmonary hemodynamics evaluated by RHC worsened with increasing RVSI. There was a gradual increase in mRAP (Figure 2A), mean pulmonary artery pressure (mPAP) and mean pulmonary artery wedge pressure (mPAWP) with increasing RVSI, whereas, there was no significant change in cardiac index (CI). As regards medications, the use of diuretic increased in proportion to RVSI. In contrast, angiotensin converting enzyme inhibitor, angiotensin II receptor blocker, angiotensin receptor-neprilysin inhibitor, mineralocorticoid receptor antagonist and sodium glucose cotransporter 2 inhibitor did not differ among the groups. Mean RAP showed a significant stepwise increase along the RVSI groups (Figure 2A, control, low RVSI and high RVSI groups) and IRVF patterns (Figure 2B, continuous, biphasic and monophasic patterns). Both RVSI and IRVF patterns showed similar associations with elevated mRAP (Figure 2A; *P* < 0.001 and Figure 2B; *P* = 0.001). In addition, RVSI showed a significant stepwise increase along the IRVF patterns (Figure 2C; *P* < 0.001). RVSI also showed a significant stepwise increase along the severity of TR (Figure 2D; *P* < 0.001). TAPSE/sPAP ratio showed a significant stepwise decrease along the RVSI groups (Figure 2E; *P* < 0.001). Furthermore, there were significant correlations of RVSI with right atrial area (*R* = 0.327, *P* < 0.001), IVC (*R* = 0.327, *P* < 0.001), TR severity (*R* = 0.197, *P* < 0.001), TAPSE (*R* = −0.173, *P* = 0.002), TAPSE/sPAP ratio (*R* = −0.330, *P* < 0.001), mRAP (*R* = 0.253, *P* < 0.001), mPAP (*R* = 0.288, *P* < 0.001) and mPAWP (*R* = 0.279, *P* < 0.001), but not with LVEF (*R* = −0.063, *P* = 0.215), LVOT VTI (*R* = −0.031, *P* = 0.553) and CI (R = −0.019, *P* = 0.769). These results suggest that elevated RVSI indicates right-sided overload rather than left-sided overload.

**Table 1:** Clinical and demographic characteristics of the study population.

	**Total (*N* = 388)**	**Control (RVSI = 0, *N* = 260)**	**Low RVSI (0 < RVSI ≤ 0.21, *N* = 63)**	**High RVSI (RVSI > 0.21, *N* = 65)**	***P*** -value^*^
**Renal Doppler ultrasonography** IRVF pattern (n, %) continuous/ biphasic/ monophasic/ unclassified	260 (67.0)/ 78 (20.1)/ 48 (12.4)/ 2 (0.5)	260 (100)/ 0 (0)/ 0 (0)/ 0 (0)	0 (0)/ 38 (60.3)/ 23 (36.5)/ 2 (3.2)	0 (0)/ 40 (61.5)/ 25 (38.5)/ 0 (0)	<0.001
**Demographics**					
Age (years)	71 (63.0–79.8)	71.0 (63.0–78.0)	69.0 (58.0–80.0)	75.0 (66.0–82.0)	0.295
Male sex (*n*, %)	225 (58.0)	155 (59.6)	36 (57.1)	34 (52.3)	0.559
Body mass index (kg/m^2^)	22.6 (20.3–25.4)	22.5 (20.4–25.4)	23.3 (20.1–24.8)	21.9 (20.2–25.8)	0.921
Systolic BP (mmHg)	116.0 (105.0–130.0)	117.0 (106.0–130.0)	113.0 (101.8–127.3)	116.0 (103.5–132.0)	0.453
Heart rate (bpm)	69.0 (60.0–80.0)	69.0 (60.0–81.8)	69.0 (60.0–77.0)	70.0 (60.5–79.5)	0.632
NYHA class III or IV (*n*, %)	111 (28.6)	67 (27.8)	16 (26.2)	28 (43.8)	0.036
Etiology (*n*, %) ischemic/ myopathy/ valvular/ arrhythmia/ pulmonary/ congenital/ others	77 (19.8)/ 92 (23.7)/ 126 (32.5)/ 40 (10.3)/ 37 (9.5)/ 7 (1.8)/ 9 (2.3)	53 (20.4)/ 65 (25.0)/ 84 (32.3)/ 25 (9.6)/ 23 (8.8)/ 5 (1.9)/ 5 (1.9)	10 (15.9)/ 16 (25.4)/ 19 (30.2)/ 7 (11.1)/ 8 (12.7)/ 1 (1.6)/ 2 (3.2)	14 (21.5)/ 11 (16.9)/ 23 (35.4)/ 8 (12.3)/ 6 (9.2)/ 1 (1.5)/ 2 (3.1)	0.979
**Comorbidities**					
CAD (*n*, %)	111 (28.6)	76 (29.2)	16 (25.4)	19 (29.2)	0.827
Atrial fibrillation (*n*, %)	139 (35.8)	79 (30.4)	25 (39.7)	35 (53.8)	0.002
Hypertension (*n*, %)	249 (64.2)	156 (60.0)	44 (69.8)	49 (75.4)	0.041
Dyslipidemia (*n*, %)	255 (65.7)	180 (69.2)	41 (65.1)	34 (52.3)	0.036
Diabetes mellitus (*n*, %)	139 (35.8)	93 (35.9)	20 (31.7)	26 (40.0)	0.623
CKD (*n*, %)	251 (64.7)	158 (60.8)	42 (66.7)	51 (78.5)	0.027
Anemia (*n*, %)	175 (45.1)	105 (40.4)	26 (41.3)	44 (67.7)	<0.001
**Laboratory data**					
BNP (pg/mL)	183.8 (77.8–396.2)	157.0 (67.3–334.5)	209.8 (76.8–501.4)	305.2 (158.2–582.6)	<0.001
Log BNP	2.26 (1.89–2.60)	2.20 (1.83–2.52)	2.32 (1.89–2.70)	2.49 (2.20–2.77)	<0.001
BUN (mg/dL)	19.0 (15.0–25.0)	19.0 (15.0–25.0)	19.0 (15.0–25.0)	21.0 (16.0–26.5)	0.285
Creatinine (mg/dL)	1.00 (0.82–1.24)	0.97 (0.79–1.19)	0.98 (0.82–1.30)	1.07 (0.86–1.32)	0.129
eGFR (mL/min/1.73 m^2^)	52.5 (40.0–64.0)	54.0 (41.3–65.0)	51.0 (42.0–64.0)	45.0 (36.0–59.0)	0.017
Sodium (mEq/L)	140.0 (138.0–141.0)	140.0 (138.0–141.0)	140.0 (139.0–142.0)	140.0 (139.0–141.0)	0.186
CRP (mg/dL)	0.20 (0.08–0.70)	0.17 (0.07–0.66)	0.24 (0.06–0.68)	0.33 (0.13–1.07)	0.035
Hemoglobin (g/dL)	12.9 (11.3–14.4)	13.2 (11.6–14.7)	12.8 (11.6–14.5)	11.7 (10.3–13.5)	<0.001
Proteinuria (-)/ (±)/ (1+)/ (2+)/ missing (n, %)	205 (52.8)/ 90 (23.2)/ 52 (13.4)/ 35 (9.0)/ 6 (1.5)	152 (59.1)/ 55 (21.4)/ 28 (10.9)/ 22 (8.6)/ 3 (1.1)	26 (41.9)/ 18 (29.0)/ 13 (21.0)/ 5 (8.1)/ 1 (1.6)	27 (42.9)/ 17 (27.0)/ 11 (17.5)/ 8 (12.7)/ 2 (3.1)	0.072
UACR	22.0 (9.0–74.3)	19.0 (9.0–59.8)	22.5 (6.8–81.0)	56.0 (14.0–149.3)	0.008
**Echocardiography**					
LV ejection fraction (%)	53.0 (34.0–63.0)	54.0 (35.0–63.0)	57.0 (32.2–63.5)	46.0 (30.1–62.0)	0.201
LVOT VTI (cm)	16.1 (13.0–20.3)	16.2 (13.0–20.2)	17.5 (13.0–22.0)	15.5 (11.6–19.2)	0.219
Left atrial volume (mL)	86.0 (65.0–120.0)	82.4 (61.1–109.3)	87.5 (68.0–117.1)	119.1 (81.8–159.9)	<0.001
Mitral valve E/e'	13.7 (9.5–19.7)	12.9 (9.1–17.6)	14.5 (10.7–20.4)	15.6 (11.2–26.4)	0.006
RA area (cm^2^)	19.0 (14.0–25.0)	15.8 (13.0–23.0)	20.0 (15.4–26.0)	23.0 (17.7–30.0)	<0.001
RV diastolic area (cm^2^)	19.4 (15.0–25.6)	17.1 (12.8–23.5)	21.2 (16.9–34.2)	20.8 (18.4–27.9)	0.032
RV systolic area (cm^2^)	11.5 (8.9–17.9)	10.2 (7.8–14.6)	13.7 (10.4–24.9)	12.4 (10.3–20.1)	0.025
RV–FAC (%)	36.0 (28.0–44.0)	38.0 (32.0–44.5)	31.2 (23.0–40.5)	34.5 (23.9–44.3)	0.039
IVC (mm)	15.0 (12.7–18.6)	14.0 (12.0–17.6)	15.7 (13.0–19.3)	18.8 (15.0–22.0)	<0.001
TR (*n*, %) none-trivial/ mild/ moderate/ severe	238 (61.3)/ 90 (23.2)/ 45 (11.6)/ 15 (3.9)	170 (65.4)/ 62 (23.8)/ 25 (9.6)/ 3 (1.2)	40 (63.5)/ 14 (22.2)/ 6 (9.5)/ 3 (4.8)	28 (43.1)/ 14 (21.5)/ 14 (21.5)/ 9 (13.8)	<0.001
TRPG (mmHg)	24.9 (20.0–33.0)	23.0 (20.0–30.0)	27.0 (21.8–35.3)	29.0 (23.0–37.0)	0.003
TAPSE (mm)	17.3 (14.5–20.4)	17.7 (15.2–21.1)	17.7 (13.7–20.1)	15.0 (12.0–18.9)	0.008
TAPSE/systolic PAP ratio (mm/mmHg)	0.50 (0.36–0.68)	0.54 (0.40–0.78)	0.45 (0.29–0.55)	0.37 (0.28–0.52)	<0.001
S' (cm/s)	9.1 (7.3–10.7)	9.7 (7.9–11.2)	7.8 (6.2–10.2)	8.7 (6.9–9.8)	0.089
**Right heart catherization**					
Cardiac index (L/min/m^2^)	2.4 (2.1–2.8)	2.4 (2.1–2.9)	2.5 (2.2–2.9)	2.2 (2.0–2.7)	0.200
Mean RAP (mmHg)	7.0 (4.0–10.0)	6.0 (3.8–9.0)	7.5 (5.3–10.0)	9.0 (6.0–11.0)	<0.001
Mean PAP (mmHg)	23.0 (18.0–31.0)	21.0 (16.3–28.8)	26.0 (20.0–34.8)	28.0 (22.0–35.5)	<0.001
Mean PAWP (mmHg)	14.0 (9.0–19.0)	13.0 (8.0–17.0)	14.0 (11.3–19.8)	19.0 (14.0–22.5)	<0.001
PVR (WoodU)	2.0 (1.3–3.2)	1.9 (1.3–2.8)	2.0 (1.5–6.2)	2.8 (1.6–5.0)	0.057
**Medications**					
β-Blocker (*n*, %)	270 (69.6)	191 (73.5)	33 (52.4)	46 (70.8)	0.005
ACE-I (*n*, %)	157 (40.5)	108 (41.5)	24 (38.1)	25 (38.5)	0.827
ARB (*n*, %)	95 (24.5)	63 (24.2)	12 (19.0)	20 (30.8)	0.300
ARNI (*n*, %)	0 (0)	0 (0)	0 (0)	0 (0)	-
MRA (*n*, %)	146 (37.6)	92 (35.4)	25 (39.7)	29 (44.6)	0.364
SGLT2 inhibitor (*n*, %)	1 (0.3)	0 (0)	1 (1.6)	0 (0)	0.075
Diuretic (*n*, %)	253 (65.2)	157 (60.4)	44 (69.8)	52 (80.0)	0.009

*RVSI, renal venous stasis index; IRVF, intrarenal venous flow; BP, blood pressure; NYHA, New York Heart Association; CAD, coronary artery disease; CKD, chronic kidney disease; BNP, B-type natriuretic peptide; BUN, blood urea nitrogen; eGFR, estimated glomerular filtration rate; CRP, C-reactive protein; UACR, urine albumin-to-creatinine ratio; LV, left ventricle; LVOT VTI, left ventricular outflow tract velocity-time integral; mitral valve E/e', early transmitral flow velocity to mitral annular velocity ratio; RA, right atrial; RV, right ventricle; RV-FAC, right ventricle fractional area change; IVC, inferior vena cava diameter; TR, tricuspid regurgitation; TRPG, tricuspid regurgitation pressure gradient; TAPSE, tricuspid annular plane systolic excursion; S', tissue Doppler-derived tricuspid lateral annular systolic velocity (tricuspid valve S'); RAP, right atrium pressure; PAP, pulmonary artery pressure; PAWP, pulmonary artery wedge pressure; PVR, pulmonary vascular resistance; ACE-I, angiotensin converting enzyme inhibitor; ARB, angiotensin II receptor blocker; ARNI, angiotensin receptor-neprilysin inhibitor; MRA, mineralocorticoid receptor antagonist; SGLT2 inhibitor, sodium glucose cotransporter 2 inhibitor*.

* *A p-value indicates statistically significance in comparison across all groups*.

**Figure 3:**
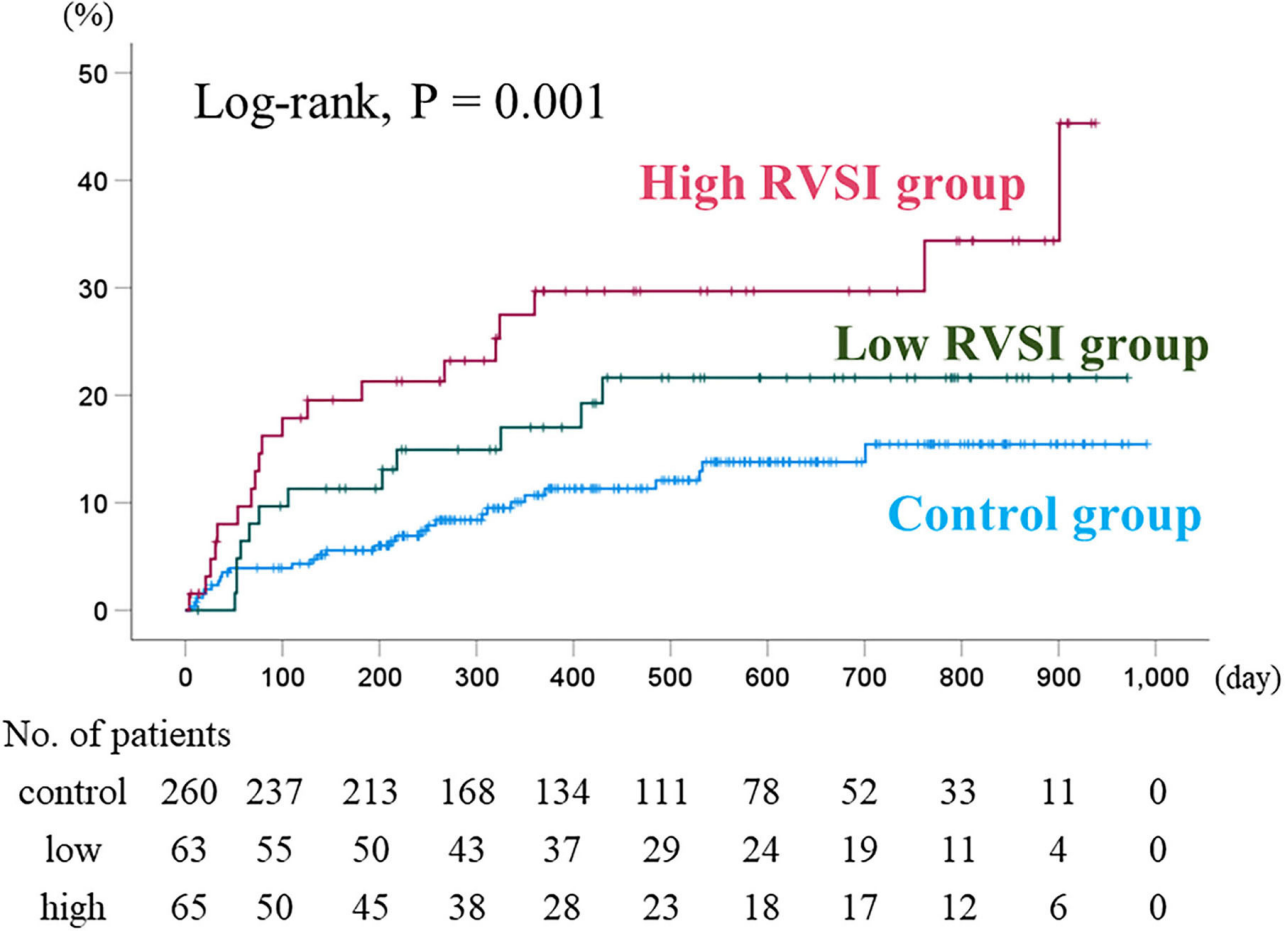
Kaplan–Meier analysis for cardiac event rates stratified by RVSI. The cumulative cardiac event rate significantly increased with increasing RVSI (log-rank, *P* = 0.001). RVSI, renal venous stasis index.

**Figure 4:**
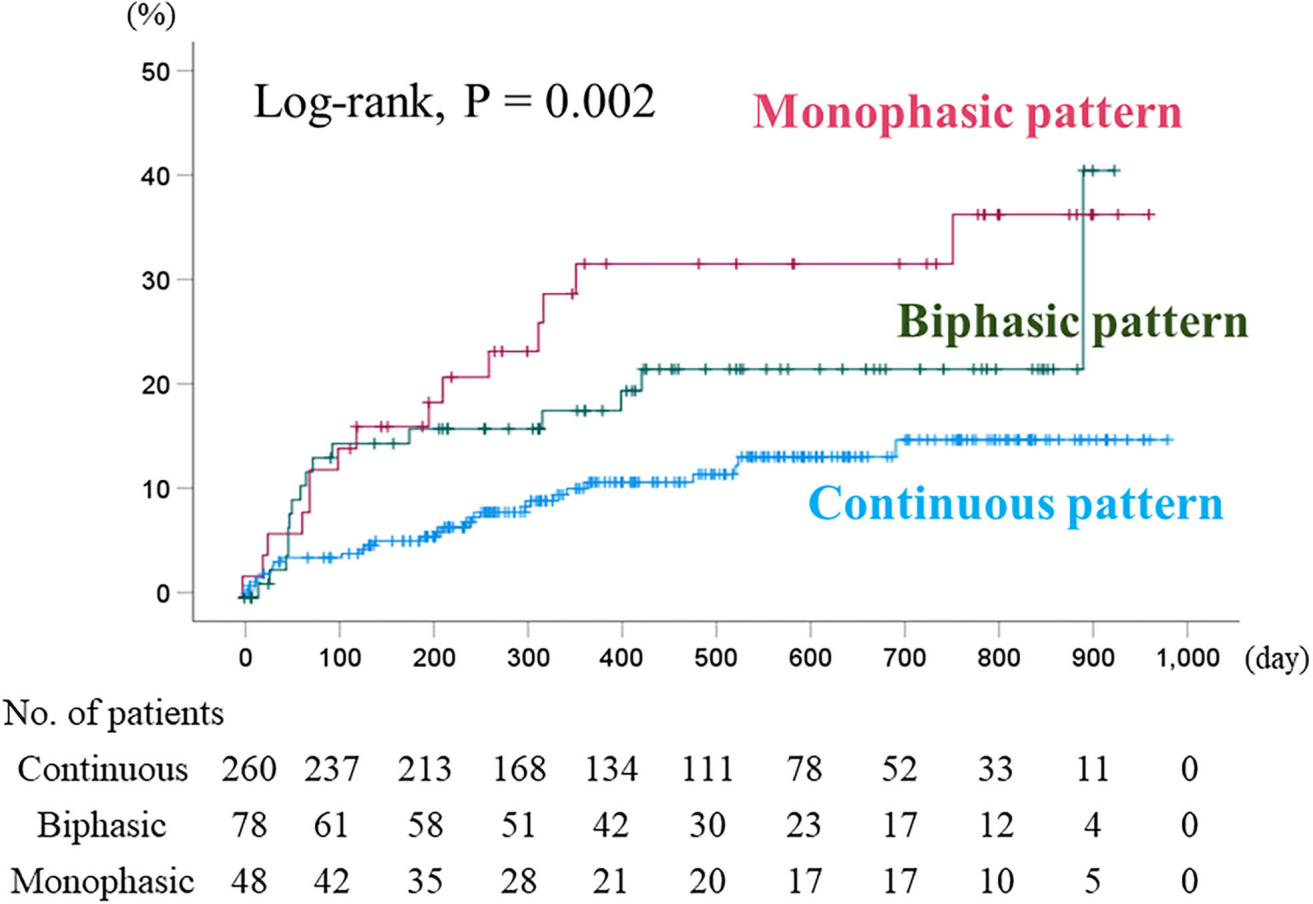
Kaplan–Meier analysis for cardiac event rate stratified by IRVF patterns. The cumulative cardiac event rate significantly increased with worsening IRVF patterns (log-rank, *P* = 0.002). Although it showed basically similar to those of RVSI classification, there were overlap and inversion between the groups with biphasic and monophasic IRVF patterns. IRVF, intrarenal venous flow.

During the follow-up period (median 412 days; range 4–991 days), cardiac events occurred in 60 patients (16 cardiac deaths and 52 worsening HF). Sixteen cardiac deaths included 14 deaths from HF and 2 deaths from ventricular fibrillation. In the Kaplan–Meier analysis, the cumulative cardiac event rate significantly increased with increasing RVSI (Figure 3, log-rank, *P* = 0.001). In the univariate Cox proportional hazard analysis (Table 2), high RVSI was associated with high cardiac event rate (high RVSI group vs. control group, hazard ratio, 2.849; 95% confidence interval, 1.596–5.087, *P* < 0.001). In the multivariate Cox proportional hazard analyses, due to the limited number of cardiac events (60 events) and to avoid overfitting, we selected univariate factors with *P* < 0.05 (i.e., age, CKD, anemia, BNP and high RVSI). After adjusting for these confounding factors, high RVSI was an independent prognostic factor (Table 2; high RVSI group vs. control group: hazard ratio, 1.908; 95% confidence interval, 1.046–3.479, *P* = 0.035). In the subgroup analysis regarding LVEF, there was no significant interactions between prognostic impact of RVSI and LVEF (*P* = 0.759), and high RVSI was associated with high cardiac event rate both in the heart failure with reduced ejection fraction group and the heart failure with preserved ejection fraction group. In the Kaplan–Meier analysis stratified by IRVF patterns, the cumulative cardiac event rate significantly increased with worsening IRVF patterns (Figure 4, log-rank, *P* = 0.002). There were overlap and inversion between the groups with biphasic and monophasic IRVF patterns.

**Table 2:** Cox proportional hazard model for predicting cardiac events.

**Cardiac events**	**Univariate**			**Multivariate**		
	**HR**	**95% CI**	***P*** **-value**	**HR**	**95% CI**	***P*** **-value**
Age	1.038	1.015–1.063	0.001	1.016	0.990–1.042	0.232
Sex (male)	0.994	0.595–1.661	0.981			
atrial fibrillation	1.225	0.731–2.055	0.441			
hypertension	1.216	0.706–2.096	0.481			
dyslipidemia	1.198	0.689–2.084	0.522			
CKD	3.345	1.693–6.608	0.001	2.174	1.056–4.476	0.035
Anemia	2.113	1.260–3.544	0.005	1.313	0.755–2.286	0.335
BNP	1.002	1.001–1.002	<0.001	1.001	1.001–1.002	<0.001
**RVSI**						
Low RVSI vs. control	1.648	0.841–3.231	0.146			
High RVSI vs. control	2.849	1.596–5.087	<0.001	1.908	1.046–3.479	0.035

*RVSI, renal venous stasis index; CKD, chronic kidney disease; BNP, B-type natriuretic peptide; HR, hazard ratio; CI, confidence interval*.

## Discussion

In this study, we investigated the relationship of RVSI assessed by renal Doppler ultrasonography with laboratory tests, echocardiography and RHC, as well as its prognostic impact in HF patients. According to a past study, PH patients with high RVSI experienced more adverse events such as worsening PH or all-cause mortality (10). However, the clinical significance of RVSI in HF patients has not been sufficiently examined.

Right ventricular function is the main determinant of symptomatology and outcome in patients with HF (23, 25, 26). Right ventricle adapts to increased afterload by increasing contractility to preserve the right ventricle–to–pulmonary circulation coupling. When this adaptation is unsustainable, the right ventricle increases preload (in other words, right ventricular end-diastolic volume) relying on Frank-Starling law (26). In normal subjects without right ventricular diastolic failure, the forward flow from the right atrium to the right ventricle is normal, because the right ventricular end-diastolic pressure remains low. However, in patients with right ventricular diastolic failure, the forward flow is impaired, because the right ventricle is stiff and right ventricular end-diastolic pressure is elevated. When the pressure in the vena cava (IVC and superior vena cava) becomes lower than that in the right ventricle, blood flow will be partially directed backward into the vena cava. This leads to elevation of RAP (29). Namely, RAP is a surrogate marker of both right ventricular diastolic function and right ventricular diastolic stiffness (30–33). Furthermore, these series of processes lead to reduction of venous return to the right ventricle, and result in loss of right ventricular stroke volume and right ventricular dysfunction. In the present study, there was a positive correlation between RVSI and mPAP (*R* = 0.253, *P* < 0.001). RVSI also showed a positive correlation with TR severity (*R* = 0.197, *P* < 0.001) and a negative correlation with TAPSE/sPAP ratio (*R* = −0.330, *P* < 0.001). With advancing right-sided heart failure, TR generally becomes more severe (34). TAPSE/sPAP ratio is a surrogate of the right ventricle–to–pulmonary circulation coupling and is affected by right ventricular diastolic stiffness (26). We consider that RVSI was mainly affected by RAP, and was also affected by right heart diastolic failure, backflow, forward flow impairment and etc. Concordant with our results, it has been reported that RAP, right ventricular dysfunction, severity of TR and TAPSE/sPAP ratio are associated with worse prognosis in HF patients (25–27, 29–32, 35–43). To the best of our knowledge, this study is the first to report that the RVSI, as a marker of right-sided overload, is associated with cardiac events in HF patients.

Cardiorenal syndrome commonly refers to the collective dysfunction of heart and kidney resulting in a cascade of feedback mechanism that damages both organs (44). Renal dysfunction is associated with adverse prognosis in HF patients (45). Given the aging population, patients with HF and CKD are likely to continue to increase due to longer cumulative exposure to common risk factors including hypertension, obesity, diabetes and vascular disorders (46). Thus, we have to understand the various mechanisms of this syndrome. Renal congestion due to elevation of RAP is one of the main conditions of cardiorenal syndrome. Renal congestion may increase interstitial pressure and reduce vessel compliance in the renal parenchymal regions due to direct compression (47–49). Intrarenal venous flow depends on interstitial pressure, intra-abdominal pressure and intravenous pressure, and shows superimposed biphasic forward velocities that peak during systole and diastole (48, 50, 51). Under physiological conditions, intrarenal veins exhibit continuous flow independent of renal function (continuous pattern). With increasing RAP, renal veins become less compliant, continuous flow becomes discontinuous flow and increasing prominence of the superimposed biphasic forward velocities (biphasic pattern). Further increases in RAP finally lead to a diastolic-only flow (monophasic pattern) (52, 53). The classification of IRVF patterns has a weakness; some patterns may be difficult to be classified. According to Table 1, there were no differences in distribution of IRVF patterns between the high and low RVSI groups. RVSI can complement the weaknesses by quantifying IRVF. We consider that quantifying RVSI can detect renal congestion more sensitively than IRVF patterns.

We considered both RVSI and IRVF pattern might be useful as markers of right sided heart failure and prognostic indicators. Regarding comparison of RVSI with IRVF patterns for predicting prognosis, our data suggest that the RVSI may be superior to IRVF patterns. Namely, although Kaplan–Meier analysis of IRVF patterns (Figure 4) showed basically similar to those of RVSI classification (Figure 3), there were overlap and inversion between the biphasic and monophasic IRVF patterns (Figure 4). Additionally, to compare the high and low RVSI groups with the same IRVF patterns about cardiac event rates, in patients with biphasic pattern, we could find that the cardiac event rate was significantly higher in the high RVSI group than in the low RVSI group in the Kaplan–Meier analysis (log-rank, p = 0.004, data not shown in the manuscript). Moreover, RVSI was reported to be more sensitive and specific predictor of the outcome than IRVF patterns in the previous study (20). Taken together, RVSI can predict outcome more sensitive than IRVF patterns, even in cases of same IRVF pattern.

## Study Limitations

There are some limitations in the present study. First, the study may be somewhat underpowered because of a single center prospective cohort study with a relatively small number of patients and a short follow-up period. Second, the present study used only variables during hospitalization and did not take into consideration changes in medical parameters or treatments after discharge. The hemodynamics of HF patients changes dynamically. Thus, future studies need to assess alterations of RVSI in response to hemodynamic changes. Third, since the attending physicians made decisions to perform RHC, there might be a potential selection bias. Therefore, the present results need to be viewed as preliminary, and further studies with a larger number of patients are needed.

## Conclusion

RVSI assessed by renal Doppler ultrasonography reflects right-sided overload and is associated with adverse prognosis in HF patients.

## Data Availability Statement

The raw data supporting the conclusions of this article will be made available by the authors, without undue reservation.

## Ethics Statement

The studies involving human participants were reviewed and approved by Ethics Committee of Fukushima Medical University. The patients/participants provided their written informed consent to participate in this study. Written informed consent was obtained from the individual(s) for the publication of any potentially identifiable images or data included in this article.

## Author Contributions

HO, KW, and YSa: conceptualization, methodology, formal analysis, investigation, writing–original draft, and visualization. AY: conceptualization, methodology, formal analysis, investigation, resources, data curation, writing–original draft, visualization, supervision, project administration, and funding acquisition. YHor, SI, MM, and YY: methodology, investigation, writing–original draft, and visualization. YSu, YI, YHot, TM, TK, MO, and AK: conceptualization, methodology, formal analysis, investigation, and writing–review and editing. YT: conceptualization, methodology, formal analysis, investigation, writing–original draft, supervision, and project administration. All authors contributed to the article and approved the submitted version.

## Funding

This study was supported in part by a grant-in-aid for Scientific Research (Nos. 20K07828 and MO20K16529) from the Japan Society for the Promotion of Science.

## Conflict of Interest

The authors declare that the research was conducted in the absence of any commercial or financial relationships that could be construed as a potential conflict of interest.

## Publisher's Note

All claims expressed in this article are solely those of the authors and do not necessarily represent those of their affiliated organizations, or those of the publisher, the editors and the reviewers. Any product that may be evaluated in this article, or claim that may be made by its manufacturer, is not guaranteed or endorsed by the publisher.
