# Supplementary material for: Renal Venous Stasis Index Reflects Renal Congestion and Predicts Adverse Outcomes in Patients With Heart Failure
Source: Front Cardiovasc Med. 2022 Mar 7;9:772466. doi: 10.3389/fcvm.2022.772466 (PMC8934863; doi:10.3389/fcvm.2022.772466)
Supplement: Supplementary file 1 [file Data_Sheet_1.PDF]

Table. Subgroup analysis for predicting cardiac events: the impact of high RVSI.

Factor	***Subgroup***	n	***HR***	***95% CI***	***P-value***	***Interaction P value***
LVEF	Reduced and mid-range EF	176	2.566	1.150–5.727	0.021	0.759
	Preserved EF	211	3.114	1.346–7.201	0.008	
